# Accuracy assessment of plant height using an unmanned aerial vehicle for quantitative genomic analysis in bread wheat

**DOI:** 10.1186/s13007-019-0419-7

**Published:** 2019-04-15

**Authors:** Muhammad Adeel Hassan, Mengjiao Yang, Luping Fu, Awais Rasheed, Bangyou Zheng, Xianchun Xia, Yonggui Xiao, Zhonghu He

**Affiliations:** 1 Institute of Crop Sciences, National Wheat Improvement Centre, Chinese Academy of Agricultural Sciences (CAAS), Beijing, 100081 China; 2 College of Agronomy, Xinjiang Agricultural University, Ürümqi, 830052 China; 3 International Maize and Wheat Improvement Centre (CIMMYT) China Office, c/o CAAS, Beijing, 100081 China; 4 Department of Plant Sciences, Quaid-i-Azam University, Islamabad, 45320 Pakistan; 5 CSIRO Agriculture and Food, Queensland Bioscience Precinct, 306 Carmody Road, St Lucia, 4067 Australia

**Keywords:** Aerial surveillance, Genomic prediction, Quantitative trait loci, *Triticum aestivum*

## Abstract

**Background:**

Plant height is an important selection target since it is associated with yield potential, stability and particularly with lodging resistance in various environments. Rapid and cost-effective estimation of plant height from airborne devices using a digital surface model can be integrated with academic research and practical wheat breeding programs. A bi-parental wheat population consisting of 198 doubled haploid lines was used for time-series assessments of progress in reaching final plant height and its accuracy was assessed by quantitative genomic analysis. UAV-based data were collected at the booting and mid-grain fill stages from two experimental sites and compared with conventional measurements to identify quantitative trait loci (QTL) underlying plant height.

**Results:**

A significantly high correlation of *R*^2^ = 0.96 with a 5.75 cm root mean square error was obtained between UAV-based plant height estimates and ground truth observations at mid-grain fill across both sites. Correlations for UAV and ground-based plant height data were also very high (*R*^2^ = 0.84–0.85, and 0.80–0.83) between plant height at the booting and mid-grain fill stages, respectively. Broad sense heritabilities were 0.92 at booting and 0.90–0.91 at mid-grain fill across sites for both data sets. Two major QTL corresponding to *Rht*-*B1* on chromosome 4B and *Rht*-*D1* on chromosome 4D explained 61.3% and 64.5% of the total phenotypic variations for UAV and ground truth data, respectively. Two new and stable QTL on chromosome 6D seemingly associated with accelerated plant growth was identified at the booting stage using UAV-based data. Genomic prediction accuracy for UAV and ground-based data sets was significantly high, ranging from *r* = 0.47–0.55 using genome-wide and QTL markers for plant height. However, prediction accuracy declined to *r* = 0.20–0.31 after excluding markers linked to plant height QTL.

**Conclusion:**

This study provides a fast way to obtain time-series estimates of plant height in understanding growth dynamics in bread wheat. UAV-enabled phenotyping is an effective, high-throughput and cost-effective approach to understand the genetic basis of plant height in genetic studies and practical breeding.

**Electronic supplementary material:**

The online version of this article (10.1186/s13007-019-0419-7) contains supplementary material, which is available to authorized users.

## Background

Plant height is an important agronomic trait and it was reduction in plant height that enabled the Green Revolution [1]. Although plant height has been reduced to around 75–80 cm for irrigated wheat with high yield potential, its control remains a very important aspect in breeding programs. Two major genes, *Rht1* (or *Rht*-*B1b*) and *Rht2* (or *Rht*-*D1b*) confer reduced plant height without detrimental effects on grain yield potential in varying environments [2]. *Rht* genes also have confounding effects on anther extrusion: a major trait for hybrid wheat production [3, 4], resistance to Fusarium head blight (FHB) [5, 6], and resistance to at least one insect pest [7]. Therefore, fine-tuning of plant height for a target environment is not only important for pure-line breeding but can also be important in hybrid wheat breeding where tallness of the male parent is required for efficient production of hybrids [8]. However, the association of *Rht*-*B1* and *Rht*-*D1* with undesirable traits, for example shortened coleoptile length, has caused wheat researcher to seek alternate dwarfing genes with less adverse effects. Recently, *Rht24* was reported as new gene for reduced plant height but affecting floral architecture and response to FHB [8, 9]. It was also reported to increase kernel weight [10]. Reports of some other reduced height genes, such as *Rht4*, *Rht5*, *Rht7*, *Rht8*, *Rht9*, *Rht12*, *Rht13*, *Rht14*, *Rht16*, *Rht18*, *Rht21*, *Rht23*, and *Rht25*, also offer other possibilities for wheat improvement [11].

Marker-assisted selection based on quantitative trait loci (QTL) or functional genes can enhance the selection accuracy and ultimately increase genetic gain in each breeding cycle [12, 13]. Wheat has determinate growth habit thus plant height progressively increases during vegetative growth until the reproductive stage. Conventionally, plant height is measured once, after anthesis when full height potential has been reached. Therefore, temporal characterization of plant height could provide a better understanding about the mechanism of plant growth and underlying genetics [14]. Quantitative methods, such as QTL analysis and association mapping, can give an insight about the genetic loci and genomic prediction analysis help in selection of genotypes with strong genetic basis for trait of interest [15, 16].

Multi-location characterization of wheat germplasm is essential to evaluate adaptability of genotypes and patterns of G × E interaction for trait stability [17]. Field-based phenotyping tends to be laborious, with high likelihood of error and represents a major bottleneck for genome-to-phenome knowledge [18]. High throughput phenotyping platforms have higher capability for high precision, non-destructive characterization of quantitative traits [19]. Recent advances in proximal remote sensing using unmanned aerial vehicles (UAV) with RGB (red, green, blue) and multi-spectral imaging have made it possible to create high throughput, cost-effective and accurate quantitative phenotyping datasets [12, 20]. UAV platforms can easily acquire multi-point data for complex traits such as biomass, normalized difference vegetation index, plant density, early emergence, rate of senescence rate, and plant height [20–25]. These platforms are low cost compared to traditional and recently advanced ground-based phenotyping platforms [25].

UAV-based plant height has been estimated using digital surface models (DSM). High correlations with ground-based reference measurements have been made for barley [21], wheat [26], poppy [27] and sorghum [28]. DSM gives information of altitude in the form of raster values. The drawbacks of previous approaches were that estimations were made of the average heights of whole canopies, including not only the heights of ears, but also the heights of lower leaves and even the elevation of bare ground patches within canopy gaps [29]. Furthermore, accurate assessment of the ground surface elevation imposes a major restriction factor data acquisition for UAV-based phenotyping of plant height in crops such as wheat with dense canopies. These limitations have made UAV-based platforms more complex and time-consuming by increasing the workload such as flights before planting and post-imaging quality control analysis [30]. This kind of data noise can adversely affect genetic analyses and genome-based selection. Previously, DSM-derived plant height data had been applied for genomic prediction in sorghum [24]. Therefore, there is a need to standardize UAV-based data for accurate and error-free characterization of plant height for quantitative genetic studies and selection of advanced lines in breeding program. To date, there is no report on the use of UAV-derived plant height data for quantitative loci analysis in wheat.

The major objectives of the present study were to (1) standardize a rapid method for plant height estimation using a UAV platform, (2) identify quantitative trait loci for plant height using UAV and ground-based measurements, and (3) assess genomic prediction accuracy for plant height in wheat.

## Materials and methods

### Germplasm and experimental design

A set of 198 doubled haploid (DH) lines derived from the cross Yangmai 16/Zhongmai 895 were used to evaluate a UAV-based platform for measuring plant height and its application in QTL analysis and genomic prediction. Yangmai 16 and Zhongmai 895 are elite varieties that have been widely cultivated in Yangtze River, and Yellow and Huai Valleys regions, respectively. Experiments were conducted during 2016–2017 and 2017–2018 at Xinxiang (35°18′0″N, 113°52′0″E) and Luohe (33°34′0″N, 114°2′0″E), both in Henan province. The DH lines and two parents were planted in randomized complete blocks with three replications (200 genotype × 3 replications) at each site. The size of each plot was 3.9 m^2^ (1.3 m × 3 m) with six rows at 0.30 cm spacing and the plant density was maintained at 270 plants/m^2^. Both sites were irrigated at same developmental stages according to local agricultural practices.

### Remote sensing campaign, mosaicking and DSM generation

An auto-operational DJI Inspires 1 model T600 (SZ DJI Technology Co., Shenzhen) carrying a Sequoia 4.0 camera (https://www.micasense.com/parrotsequoia/) was used for aerial imagery. Sequoia has a 16-megapixel RGB camera and 4 monochrome sensors (NIR, Red, Green and Red-edge). Flight missions over the targeted field were controlled by flight planning software Altizure DJI version 3.6.0 (https://www.altizure.com). Images were acquired in sunny conditions from 30 m altitude while maintaining 85% forward and 85% side overlapping between images to ensure enough ground sampling distance. Pix4D Mapper (PIX4d, Lausanne, Switzerland) (https://pix4d.com/) was used for orthomosaic and DSM generation using world geographic coordinates of GCPs as previously reported by Hassan et al. [20]. Pix4D has the advantage of auto-processing in feature point matching and point cloud generation. All correspondence between overlapping images estimated from their geographical coordinates and pixels were used to detect the accuracy of matching points to minimize spaces between point clouds. The image resolution or ground sampling distance at 30 m was 2.5 cm/pixel.

### DSM evaluation and plant height model (PHM) development

**Fig. 1:**
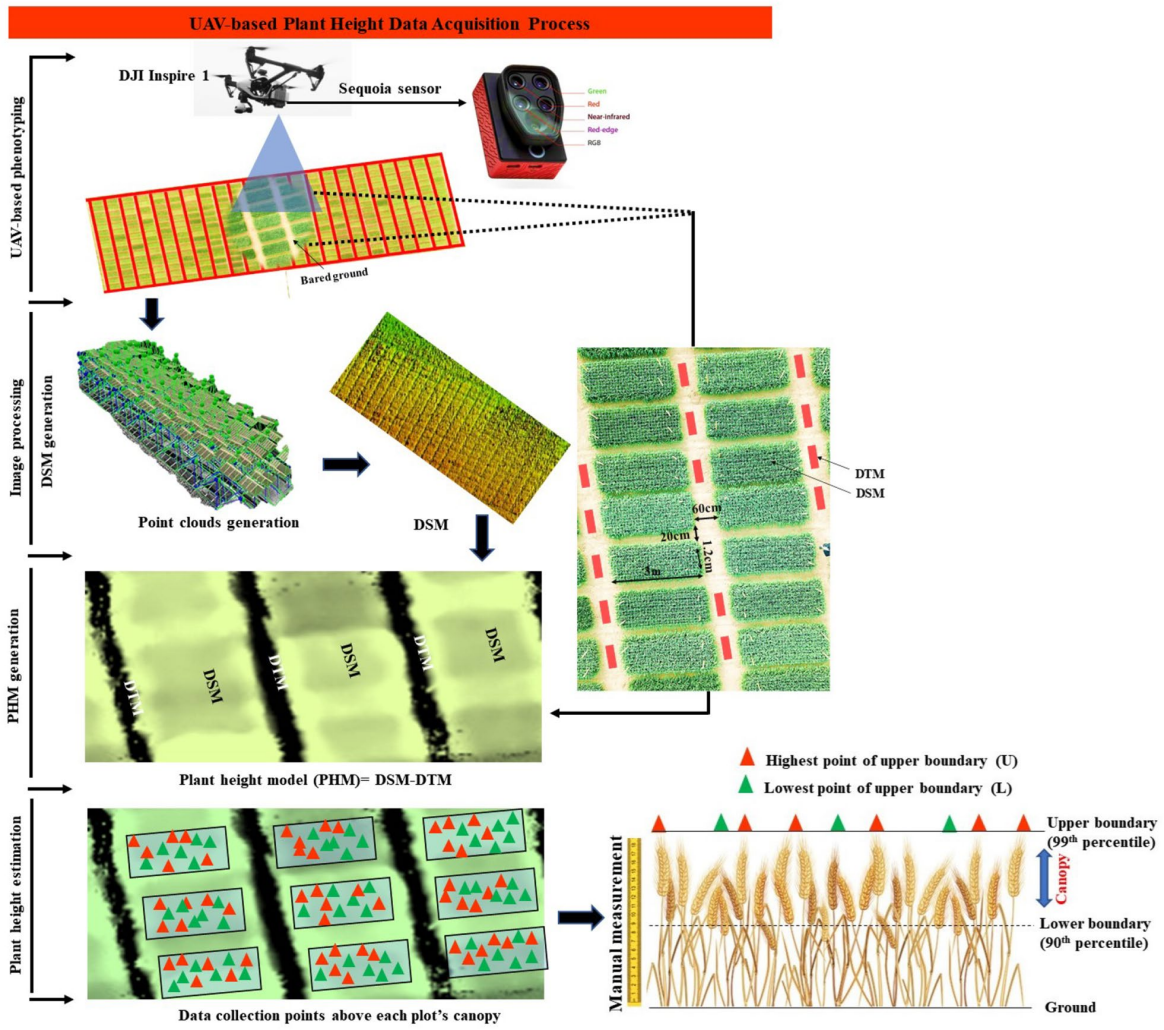
Phenotyping pipeline for estimation of plant height using UAV platform. *DTM* digital terrain model, *DSM* digital height model, *PSM* plant surface model, *UAV* unmanned aerial vehicle

As wheat canopies are relatively dense at maturity, there are lower possibilities of error in detecting bare ground patches within the canopy, especially if plants densities are maintained at 270 plants/m^2^. For more accuracy, ortho-mosaic images with Red and Green bands were used to classify the vegetation and bare ground soil [27]. Visual classification of bare soil patches and separation between plots were also done by RGB images. DSM generation was based on the World Geodetic System (1984), which does not reflect the actual height of canopies. The digital terrain model (DTM) was generated through raster values of bare ground along the edges of each plot; this gave information on the altitude of the ground surface [21]. For this, polygon shapes were sketched on bare ground surfaces across the experimental area to determine the lowest and highest ground elevation points in each zone, to minimise overall surface curvature using QGIS 1.18.15 (www.qgis.org). The PHM was calculated by subtracting the DTM from the DSM (Fig. 1).

1 $${\text{PHM}} = {\text{DSM}} - {\text{DTM}}$$

### Estimation and validation of UAV-based plant heights

After construction of the PHM, a workflow program reported by Hassan et al. [20] was followed for segmentation of the PHM into specific genotypes representing plots by sketching polygon shapes using QGIS 1.18.15 (www.qgis.org). For precise segmentation, ortho-mosaic images generated sequentially with DSM were used for segmentation. In order to avoid over-lapping of plants from adjacent plots, plant heights were estimated from a trimmed section of the plots to overcome expected data noise at the plot margins. UAV-based plant height was averaged from pixel values obtained at the highest and lowest points in the upper boundary of the canopy to avoid detection of low pixel values from lower canopy boundaries. The lower boundary of the canopy might include the elevation of gaps between plants and leaves. Lower and upper elevations of each plot from PHM were estimated by zonal statistics of polygon shapes using QGIS 1.18.15 (www.qgis.org). Small polygon shapes within each plot were sketched randomly to obtain upper and lower boundaries of the canopy top assuming a 10 cm difference while rejecting the extreme lower values that could not be spike height. Individual plant heights were calculated as the mean of randomly estimated upper and lower boundaries of the canopy and used for validation and statistical analysis (Fig. 1).

2 $${\text{H}} = {\text{average}}\,({\text{U}} + {\text{L}})$$

H is plant height estimated from PHM, where U is the highest point and L is the lowest point of the upper boundary of the canopy at specific location.

UAV-based plant height was validated through ground-based measurements using a ruler at mid-grain fill. Plant height was averaged from 10 plants of each plot representing a DH line. A total 600 of plots were measured in 2 days at each experimental site. Average height error was calculated as the difference between ground measurements and plant height estimated from the UAV platform. The root means square error (RMSE) was also calculated along with the regression fit for validation of UAV platform measurements.

### SNP genotyping, QTL analysis and genomic prediction

The Yangmai 16/Zhongmai 895 DH population and parents were genotyped at Capital Bio Corporation (Beijing, China; http://www.capitalbio.com) using the commercially available Affymetrix wheat 660 K SNP array.Previously, This array was used for genome-wide QTL mapping studies [30–32]. IciMapping 4.0 was used for linkage map construction using Kosambi mapping approach. Inclusive composite interval mapping-additive (ICIM-ADD) method was used for the QTL analysis at LOD threshold of 2.5 [33]. To assess the accuracy of identification of QTL from UAV-based remote sensing, we cross-validated our results with ground truth data obtained at mid-grain fill. For this, the averaged data from 2 years (2016–2017 and 2017–2018) at both experimental sites was used for quantitative genomic analysis. For temporal assessment of genomic variation, plant height was phenotyped at booting and mid-grain fill. QTL with overlapping confidence intervals were considered to be the same. Differences between the phenotypic variances explained by QTL from both data sets were detected as validation for UAV-based QTL.

We evaluated whether ground-based measurements can be replaced by UAV-based remote sensing for future genomic prediction of yield-related traits. For this, rrBLUP (http://cran.rproject.org/web/packages/rrBLUP/index.html) was used to detect the genomic prediction accuracy of UAV-based plant height by comparison with ground-based reference data. To estimate genetic values for traits measured across environments, the following model was used for genomic best linear unbiased prediction (G-BLUP);

3 $$y_{i} = \mu_{i} + {\text{x}}_{\text{g}} + \varepsilon_{i} ,$$

where phenotypes are viewed as the sum of a random effect representing genomic signals (*u*_*i*_), marker effects (x_g_) and a model residual (*ε*_*i*_) [34].

We cross-validated UAV-based data through estimating predication accuracy by removing markers and chromosomes linked with major plant height reducing genes.

### Statistical analysis

Linear regression was calculated to evaluate the relationship between UAV-based plant height and ground-based manually measured data. A mixed linear model was used to test the significance (*P *≤ 0.05) of variation at among DH lines, environments and effects of their interactions for both data sets by the following general model;

4 $$Y = X\beta + Z\mu + \varepsilon$$

where Y is the response demonstrated by fixed (β) and random (μ) effects with random error (ε) and X and Z indicate fix and random effects, respectively.

Furthermore, for better understanding of G × E interaction combined heritabilities across environments were calculated:

5 $$h^{2} = \sigma_{g}^{2} /\left( {\sigma_{g}^{2} + \sigma_{ge}^{2} /{\text{r}} + \sigma_{\varepsilon }^{2} /{\text{re}}} \right)$$

where *σ*_*ge*_^*2*^ is genotype × environment interaction variance, e is number of environments and r indicates total replicates for each genotype [35]. The R packages such as “lme4” (https://CRAN.R-project.org/package=lme4) and “car” (https://CRAN.R-project.org/package=car) were used for all statistical analysis [36].

## Results

### Accuracy assessment of UAV-based plant height

**Fig. 2:**
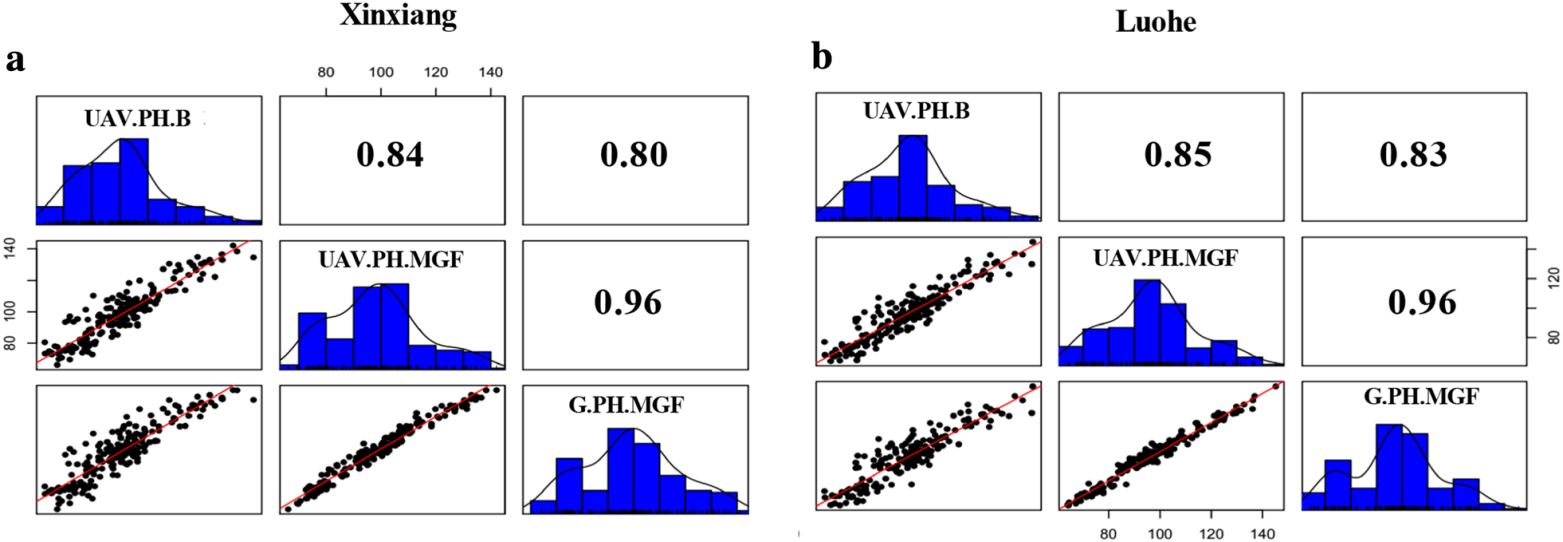
Regression plots, histograms and *R*^2^ values between UAV-based plant height at two developmental stages and ground measurements taken from two experiment locations (**a** Xinxiang and **b** Luohe). *B* booting, *G* ground, *PH* plant height, *MGF* mid grain fill, *UAV* unmanned aerial vehicle

**Fig. 3:**
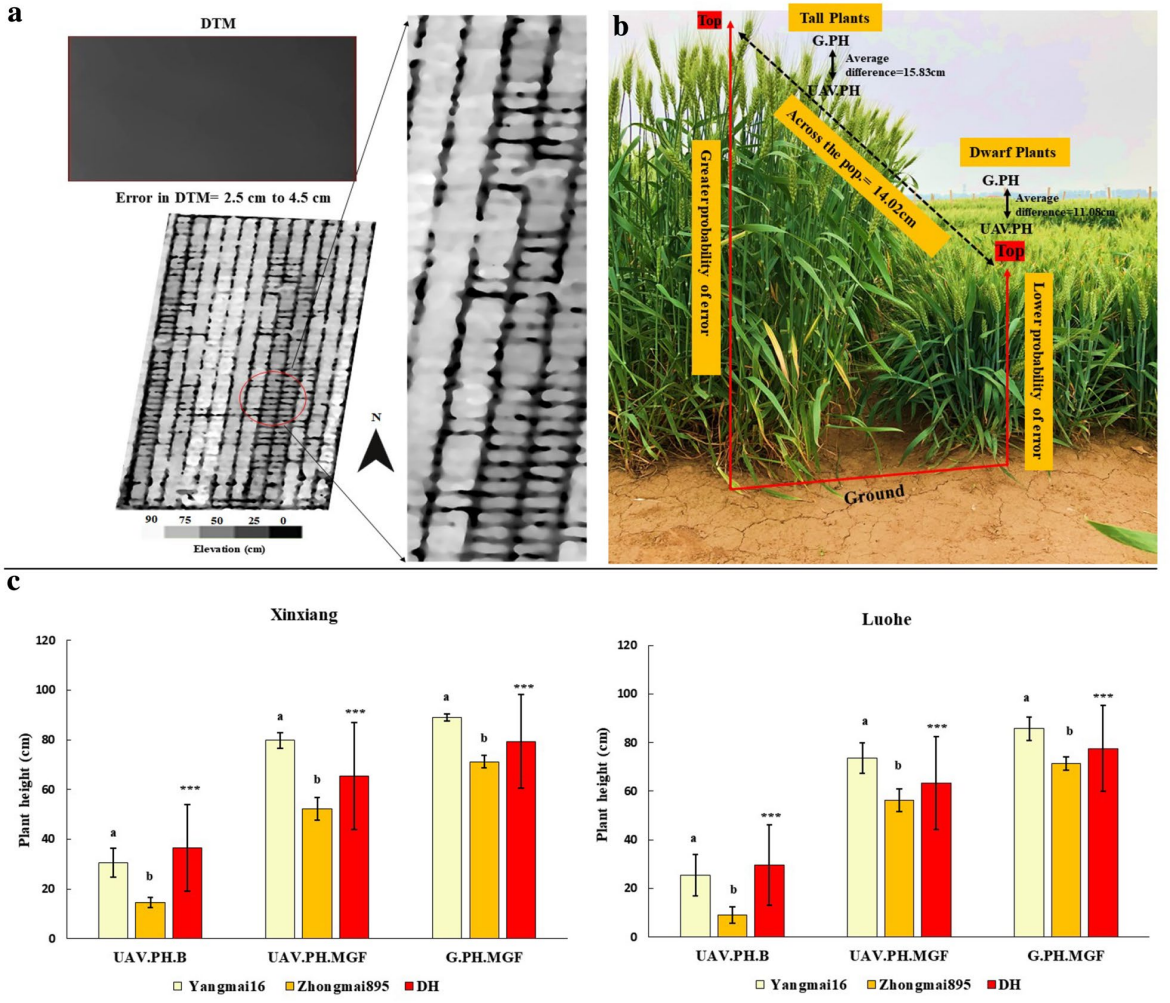
**a** Estimated probability of error between UAV and ground-based data sets for plots consisted dwarf and tall plants, **b** impact of major genes on plant height across the DH population, and **c** averaged plant height of Yangmai16, Zhongmai895 and DH. Error bars indicate standard deviation; lowercase letters indicate significant difference between parent cultivars; *. **, *** indicate significant differences among DH lines. *B* booting, *DH* doubled haploid lines, *DTM* digital terrain model, *G* ground, *MGF* mid-grain filling, *PH* plant height, *UAV* unmanned aerial vehicle

Regression analysis showed high *R*^2^ values (0.96) at both sites between UAV-based and ground-based plant height measurements at the mid-grain fill stage (Fig. 2). High correlations (*R*^2^= 0.84–0.85 and 0.80–0.83) were also obtained between booting and mid-grain fill from UAV and ground-based data sets, respectively. An accurate DTM with low error noise ranging from ± 3.5 to 4.5 cm across both sites was generated from the spaces adjacent to each plot (Fig. 3a). UAV-based single plant height was measured instead of whole canopy height through averaging highest points randomly detected from the canopy. Plant height was under-estimated but without probability of noise due to avoidance strategy for lower boundary of canopy and bare ground estimation. The average difference between predicted plant height from UAV and that observed from ground measurement was approximately 14.02 cm with a root mean square error (RMSE) of 5.75 cm across sites. Chances of error probability in estimation of UAV-based plant height were on average higher (15.83 cm) from plots with higher canopy elevations from ground level as compared to low elevation plots (11.08 cm)(Fig. 3b).

### Phenotypic variation

The average ground measurement-based plant heights of Zhongmai 895 and Yangmai 16 were 71.11 and 85.66 cm, respectively. While UAV-based plant heights of parents and DH lines are given in Fig. 3c. Plant height showed continuous variation across the DH population and followed normal distributions at both growth stages (Fig. 2). Both UAV and ground-based data sets showed similar patterns of phenotypic variation among genotypes and G × E interaction at the mid-grain fill stages (Table 1). Significant variation (*P *< *0.*0001) among the DH lines was also observed at the booting stage from the UAV data set. The standard deviation was 20.44 cm for UAV and 18.29 cm for ground-based data in DH population. Heritabilities were very high at both developmental stages, i.e. 0.92 at booting and 0.90–0.91 at mid-grain fill for UAV and ground-based plant height, respectively (Table 1).

**Table 1:** Summary of statistics for both plant height data sets and developmental stages

	Yangmai 16 and Zhongmai 895			DH population		
	UAV.PH.B	UAV.PH.MGF	G.PH.MGF	UAV.PH.B	UAV.PH.MGF	G.PH.MGF
SD	10.05	12.62	8.88	17.26	20.44	18.29
G (F.value)	431.21*	78.01*	58.24*	33.99***	43.39***	77.24***
E (F.value)	46.35*	0.19	0.54	25.61***	27.93***	47.55***
G × E (F.value)	0.06	3.74	0.80	0.59	1.56	1.52
*h* ^2^	0.92	0.91	0.93	0.92	0.90	0.91

*B* booting, *MGF* mid-grain filling, *PH* plant height, *UAV* unmanned aerial vehicle

* *P * <  0.05, ** *P * <  0.001 and *** *P * <  0.0001

### Identification of QTL and their impact on phenotype

**Fig. 4:**
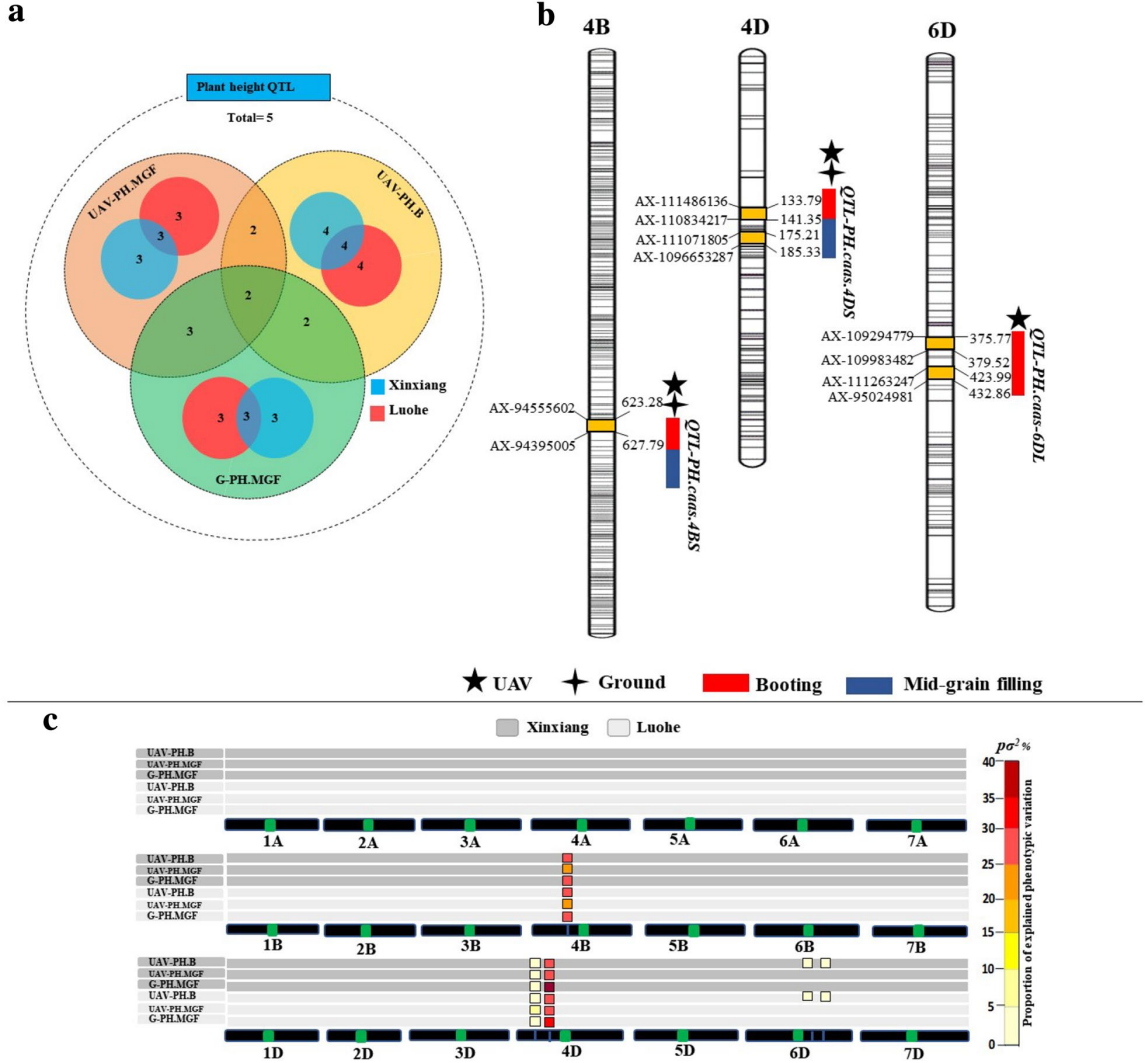
**a** Total and common QTL among two phenotyping data sets, developmental stages and environments. Numbers show the QTL in data sets, developmental stages and sites, **b** location of QTL with markers, **c** Comparison of phenotypic variance explained by QTL detected from UAV and ground-based data sets at two experimental sites. Squares with different colours show proportion of phenotypic variations explained by QTL detected in particular data set and sites on chromosomes 4B, 4D and 6D. Green spots represent the centromeres. *B* booting, *G* ground, *MGF* mid-grain fill, *UAV* unmanned aerial vehicle

Identification of QTL was performed using UAV-based phenotypic data collected at the booting and mid-grain fill stages and validated through ground truth data at mid-grain fill. Five QTL were identified from UAV and ground-based phenotypic data sets at both developmental stages and sites (Fig. 4a). Stable major QTL on chromosomes 4B and 4D were identified from UAV-based plant height data and were also detected with ground-based reference data across sites (Fig. 4b and Additional file 1: Table S1). These two QTL significantly reduced plant height in the DH population at both developmental stages (Fig. 3b). Genotypes of the DH lines are given in Additional file 1: Table S2. Another two QTL for plant height on chromosome 6D identified at the booting stage from UAV-based data explained 9.0–10.2% of phenotypic variation (Fig. 4c and Additional file 1: Table S1).

### Validation of UAV-based QTL results

**Fig. 5:**
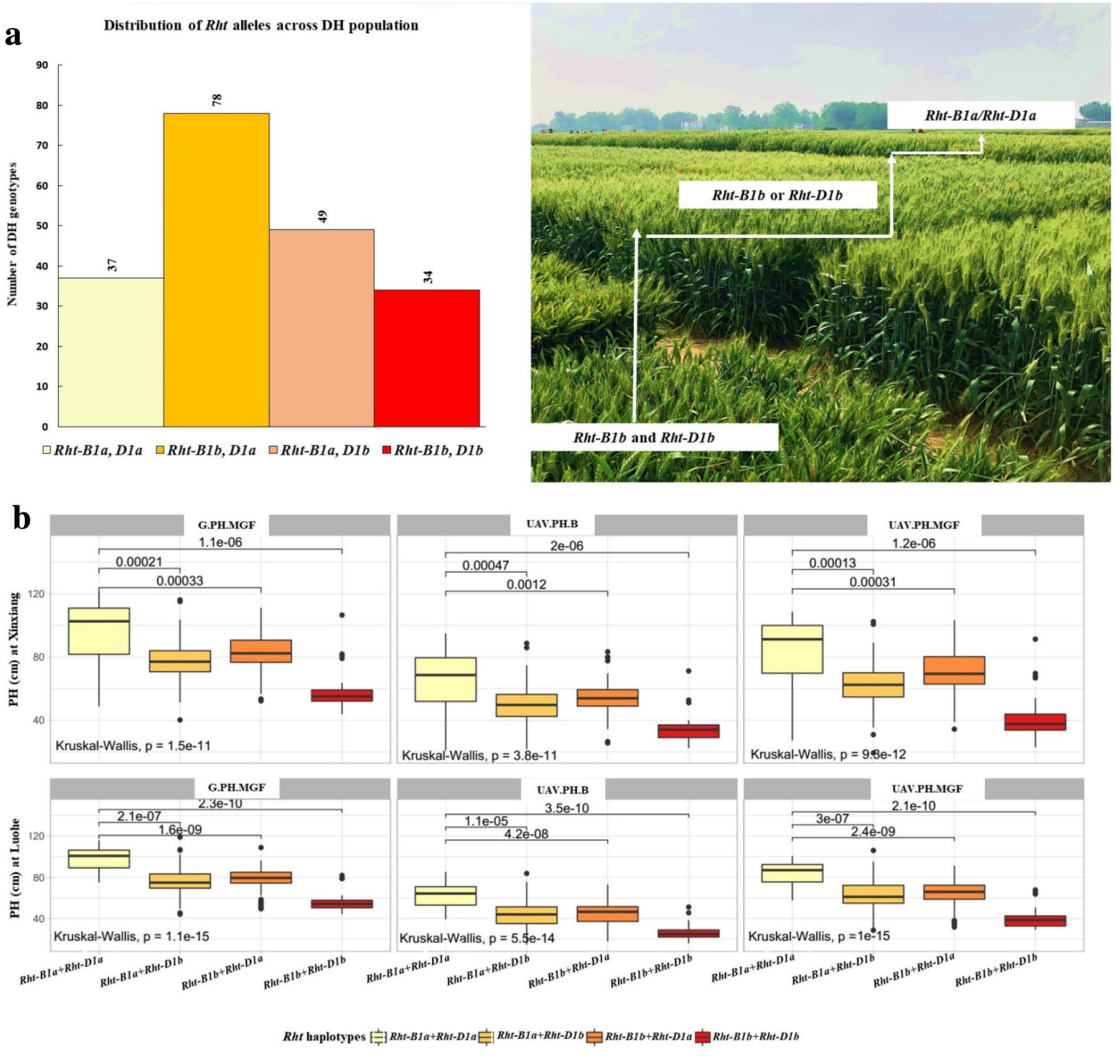
**a** Distribution of *Rht* genotypes across the DH population and **b** validation of UAV-based data set through emulating impact of these alleles using UAV-based phenotype data. *B* booting, *G* ground, *MGF* mid-grain fill, *UAV* unmanned aerial vehicle

For validation of QTL predicted from UAV-based plant height data, their contribution to phenotypic variance was compared with ground truth results at mid-grain fill. The phenotypic variances explained by the QTL located on chromosomes 4B and 4D were almost the same for both data sets, i.e. 61.3% for UAV-based and 64.5% for ground-based data with very high heritabilities of 0.90 and 0.91, respectively (Fig. 4c). These two major QTL were also identified for plant height at the booting stage, explaining 73.1% of phenotypic variance for the UAV-based data. The QTL on chromosomes 4B and 4D corresponded to reduced plant height alleles *Rht*-*B1b* and *Rht*-*D1b*, respectively. Gene-specific KASP markers (*Rht*-*B1_SNP* and *Rht*-*B1_SNP*) for *Rht*-*B1b* and *Rht*-*D1b* confirmed these results. Distributions of these alleles in the DH population are given in Fig. 5a and Additional file 1: Table S1. The QTL identified on chromosome 6D from UAV-based observations at booting showed a similar trend in variation and explained 1.50 and 1.97% of the total variation in plant height at each site (Fig. 4c). Accuracy of booting stage data for plant height was validated with markers for the *Rht*-*B1b* and *Rht*-*D1b* alleles (Fig. 5b).

### Genomic prediction accuracy of UAV-based data set

**Fig. 6:**
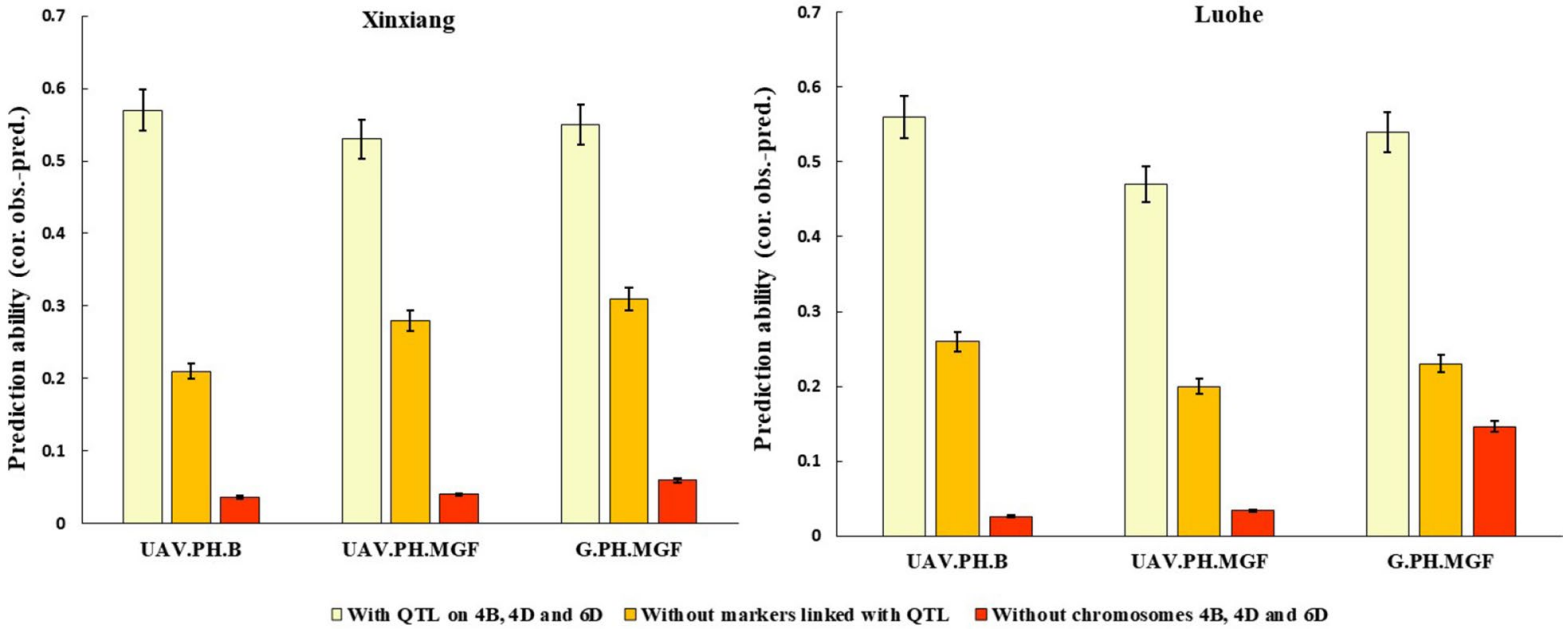
Validation of genomic prediction ability of UAV-based plant height data through with and without detected QTL at booting and mid-grain fill, as well as comparison with ground truth data at both sites. *B* booting, *G* ground, *MGF* mid-grain fill, *UAV* unmanned aerial vehicle

Genomic prediction accuracy was calculated through correlations between genetically estimated breeding values observed from a training population and was then tested by cross validation. Our results provided further validation of the accuracy of UAV-based plant height by showing similar trend regarding genomic prediction ability for both UAV and ground-based data. The correlations between observed and predicted genomic values for UAV and ground-based data sets ranged from *r *= 0.47–0.53 for UAV-based plant height at mid-grain fill, but slightly lower than ground-based truth observations of 0.54 and 055 across sites. Genomic prediction accuracy was higher ranging from *r *= 0.56–0.57 at booting when estimated from UAV-based plant height data. Prediction accuracy was significantly reduced to *r *= 0.20 and 0.31 when markers linked with QTL on chromosomes 4B, 4D and 6D were removed. Genomic prediction ability fell to 75% and 95% when all markers on chromosomes 4B, 4D and 6D were removed. (Fig. 6).

## Discussion

### Accuracy and phenotypic variations in UAV-based plant height

UAV is a promising platform to predict time-series development of crop canopies, and further use this data to understand the genetic basis of phenotypic variation [28]. Previously some studies have been reported different workflows for the estimation of plant height using UAV platform [21, 26, 28] The UAV-platform requires far fewer images and less computing capacity to construct the digital elevation model compared to ground-based imaging platforms [37]. Ground-based LiDAR technology has been reported more accurate [38], but it has some limitations such as in coving large and multilocational trials. Aerial estimation of plant height could be error-prone due to low efficiency in pre- and post-imagery processing methods such as altitude of imaging platform, accuracy in DTM construction, and height extraction strategy from images [21, 28, 29]. High altitude of the UAV flight is likely to generate low pixel resolution of images casing increased data noise. UAV flights were taken at low altitude (30 m) to minimise probability of error due to low pixel numbers. DTM gives information about the elevation of the ground surface. DTM accuracy is an important factor, and low accuracy in DTM can lead to high over- or under-estimations of canopy elevation [21, 27]. The precision in estimating depends on number and distribution of bare ground patches across experimental sites if the terrain is to geographically variable. In crops with dense canopies like wheat, it is difficult to generate accurate DTM from DSM images at later growth stages acquiring time-points to develop PHM from single flights. Terrain and distribution of bare ground can be handled through better experimental design and management. Our trial field was well managed with enough spacing between and along the plots to be used to estimate ground elevations across the field. DTM generated from both experimental sites at booting and mid-grain fill had low errors varying from ± 3.5 to 4.5 cm, similar to a previous report on a poppy crop [27] (Fig. 3b). It also reduced the computing load and time required for pre-planting flights to generate DTM of bare fields as done in other reports [24, 27, 28]. Our method also overcame the problem of data noise in height extraction from PHM due to the detection of lower parts of the canopy such as elevation of leaf from gaps between plants. Using this method, height of a single plant from a particular position of the experimental plot can be measured even in the case of a thin canopy. Higher correlations (*R*^2^= 0.96; 5.75 cm RMSE) were estimated between ground and UAV data sets at mid-grain fill. Our results were better than previous reports where correlations were slightly lower between UAV-derived plant height and reference observations (0.85–0.90) in wheat and barley [26, 29] (Fig. 2). This was due to the better strategy of measuring pixel values from the highest points of the imaging to be the upper boundary of the canopy rather than mean values from the whole canopy as previously done in wheat, barley and sorghum [21, 24, 26]. Both data sets showed transgressive segregation among DH lines relative to the parents with significant phenotypic variation and high heritability. Moreover, high heritability and no significant G × E allowed detection of stable quantitative loci for plant height.

### UAV-based QTLs and their effects on phenotype

Height reducing homoeoalleles *Rht*-*B1* and *Rht*-*D1* on the short arms of chromosomes 4B and 4D are GA-insensitive and major plummeting factor for wheat height by reduced GA response mechanism [39, 40]. Plant height in wheat is a developmental trait and the genetic basis underlying for its development over time is still being unmasked from a number of potential quantitative loci [11]. *Rht*-*B1b* and *Rht*-*D1b* were already reported in parent cultivars Yangmai16 and Zhongmai895, respectively [41]. UAV-based plant height accuracy was confirmed by identification of QTL corresponding to these *Rht* genes, high correlations between ground truth data and UAV-based data sets, and consistent identification of the same QTL in both UAV-based and ground-based datasets (Fig. 4). UAV-based phenotype data successfully verified the dynamic presence of these two major genes as previously reported by Zhang et al. [41]. Two new QTL with minor phenotypic effect of 1.50–1.97% was identified on chromosome 6D using UAV-based booting data from both sites. QTL were also identified 6D at under heat and drought condition which help plant for adaptation without confounding agronomic effects [42]. While in our study, these QTL might be involved in seedling vigour, but further validation is required. The QTL on chromosome 6D at booting is likely to affect the plant growth. The phenotypic validation of *Rht*-*B1* and *Rht*-*D1* on plant height measured by UAV confirmed the accuracy of this platform and proved that UAV has potential for genetic studies.

### Accuracy of UAV-based QTL

In quantitative genetics, erroneous phenotypic data is a major bottleneck [19]. Probability of error in UAV-based data can influence the QTL analysis and other genomics studies. In our study, accuracy of QTL detected from both data sets was also validated from multi-location trials. The identification of chromosome 4B and 4D QTL underpinning plant height was consistent across sites (Fig. 4b and Additional file 1: Table S1). Similarly, QTL with less phenotypic variation ranging 1.50–1.97% at booting was also consistent at both sites confirming the accuracy of the UAV-based platform for reliable quantitative genomic analysis. The new QTL on chromosome 6D identified using UAV-based data indicated that the UAV platform was effective in detecting genetic variation. These results indicated the potential high efficiency of UAV-based remote sensing for major QTL identification as well as temporal genetic dissection of traits.

### Accuracy of UAV-based data for genomic prediction

Genomic prediction is regarded as a relatively new breeding strategy to better exploit quantitative variation in crop breeding and in increasing selection accuracy by optimization of resource allocation in breeding programs [13, 43]. In revolutionizing phenotyping platforms for capture of data at lower cost, accuracy for true genomic selection cannot be compromised [44]. Therefore, UAV platforms have potential to contribute in enhancement of genomic prediction accuracy cos-effectively. Rutkoski et al. [44] used UAV-based multispectral secondary traits and reported their high prediction accuracy (*r *= 0.41–0.56) for traits related to grain yield in wheat. Here we demonstrate the use of plant height data captured by a UAV-based aerial platform for high accuracy genomic selection. Similar trends in prediction ability were obtained with and without consideration of the QTL across the data sets. The prediction accuracy declined as markers linked with the QTL were excluded in both data sets. However, remaining genome-wide SNPs predicted accuracy ranged from *r *= 0.20–0.31 (Fig. 6). Our results indicated the presence of an additional gene with minor effect that was not detected in earlier QTL mapping. Our findings also indicate that the use of UAV platforms for genomic selection of quantitative traits could improve prediction ability by continuous capture of cost-effective phenotypic data from multiple environments.

## Conclusions

This study describes a UAV-based method for plant height estimation in wheat and its application in quantitative genomic analysis and functional gene characterization. Traditionally, plant height is measured only once, despite the fact that progression to final plant height may differ among genotypes. Our UAV-based approach facilitates rapid, cost-effective, high-throughput capture of plant height data at different growth stages. High *R*^2^ between UAV and ground-based data sets indicated that UAV-platforms could be used for quantitative genomic analysis. This technique can also be applied in practical breeding after adjustment of UAV data according to the average difference (in this case, 14.03 cm) calculated between UAV and ground reference observations. The potential of UAV-based high throughput plant height phenotyping not only reduces the labour costs but is also capable of providing time-lapse reproducible data from large breeding trials to identify the underlying genetics and permit genomic selection for complex traits such as plant height.

## Additional file

**Additional file 1: Table S1**. QTLs identified at booting and mid-grain fill from both data sets. **Table S2**. Details of *Rht* alleles across the DH population and parent cultivars.
