# Supplementary material for: Accuracy assessment of plant height using an unmanned aerial vehicle for quantitative genomic analysis in bread wheat
Source: Plant Methods. 2019 Apr 15;15:37. doi: 10.1186/s13007-019-0419-7 (PMC6463666; doi:10.1186/s13007-019-0419-7)
Supplement: Supplementary file 1 — Additional file 1: Table S1. QTLs identified at booting and mid-grain fill from both data sets. Table S2. Details of Rht alleles across the DH population and parent cultivars. [file 13007_2019_419_MOESM1_ESM.doc]

**Accuracy assessment of plant height using an unmanned aerial vehicle for quantitative genomic analysis in bread wheat**

**Muhammad Adeel Hassan^1^, Mengjiao Yang^1, 2^, Luping Fu^1^, Awais Rasheed^1, 3, 4^, Bangyou Zheng^5^, Xianchun Xia^1^, Yonggui Xiao^1, *^ and Zhonghu He^1, 3, *^**

^1^Institute of Crop Sciences, National Wheat Improvement Centre, Chinese Academy of Agricultural Sciences (CAAS), Beijing 100081, China

^2^College of Agronomy, Xinjiang Agricultural University, Urumqi 830052, China

^3^International Maize and Wheat Improvement Centre (CIMMYT) China Office, c/o CAAS, Beijing 100081, China

^4^Department of Plant Sciences, Quaid-i-Azam University, Islamabad 45320, Pakistan

^5^CSIRO Agriculture and Food, Queensland Bioscience Precinct, 306 Carmody Road, St Lucia 4067, Australia

*Authors for correspondence: Yonggui Xiao, Email: xiaoyonggui@caas.cn, and Zhonghu He, Email: zhhecaas@163.com

**Additional file**

**Table S1**: QTLs identified at booting and mid-grain fill from both data sets

| **Site** | **Platform** | **Stage** | **QTL^a^** | **Chr.^b^** | **Marker Interval** | **Physical Interval.^c (^cM)** | **LOD^d^** | **PVE^e^ (%)** | **Add^f^** |
| --- | --- | --- | --- | --- | --- | --- | --- | --- | --- |
| Xinxiang | UAV | B | ***QPH.caas-4BS*** | 4B | AX-94555602-AX-94395005 | 623.2-627.7 | 30.17 | 34.25 | 8.93 |
|  |  |  | ***QPH.caas-4DS*** | 4D | AX-110518080-AX-110834217 | 134.5-141.3 | 6.16 | 5.06 | -3.7 |
|  |  |  | ***QPH.caas-4DS*** | 4D | AX-111071805-AX-109665328 | 175.2-185.3 | 30.2 | 36.65 | -9.05 |
|  |  |  | ***QPH.caas-6DL*** | 6D | AX-109294779-AX-109983482 | 383.7-391.1 | 2.66 | 1.98 | -2.31 |
|  |  |  | ***QPH.caas-6DL*** | 6D | AX-111263247- AX-95024981 | 423.9-432.8 | 3.649 | 1.50 | -2.44 |
|  |  | MGF | ***QPH.caas-4BS*** | 4B | AX-94555602-AX-94395005 | 623.2-627.7 | 36.53 | 29.89 | 9.43 |
|  |  |  | ***QPH.caas-4DS*** | 4D | AX-111486136-AX-110834217 | 134.5-141.3 | 5.22 | 2.77 | -2.8 |
|  |  |  | ***QPH.caas-4DS*** | 4D | AX-111071805-AX-109665328 | 175.2-185.3 | 37.65 | 36.98 | -10.29 |
|  | Ground |  | ***QPH.caas-4BS*** | 4B | AX-94555602-AX-94395005 | 623.2-627.7 | 27.98 | 27.46 | 10.06 |
|  |  |  | ***QPH.caas-4DS*** | 4D | AX-111486136-AX-110834217 | 134.5-141.3 | 5.69 | 4.29 | -3.88 |
|  |  |  | ***QPH.caas-4DS*** | 4D | AX-111071805-AX-109665328 | 175.2-185.3 | 30.75 | 35.73 | -11.27 |
| Luohe | UAV | B | ***QPH.caas-4BS*** | 4B | AX-94555602-AX-94395005 | 623.2-627.7 | 30.4 | 34.17 | 9.34 |
|  |  |  | ***QPH.caas-4DS*** | 4D | AX-110518080-AX-110834217 | 134.5-141.3 | 7.05 | 3.18 | -3.56 |
|  |  |  | ***QPH.caas-4DS*** | 4D | AX-111071805-AX-109665328 | 175.2-185.3 | 31.77 | 38.74 | -9.74 |
|  |  |  | ***QPH.caas-6DL*** | 6D | AX-109294779-AX-109983482 | 383.7-391.1 | 2.76 | 1.97 | -2.41 |
|  |  |  | ***QPH.caas-6DL*** | 6D | AX-111263247- AX-95024981 | 423.9-432.8 | 3.89 | 1.61 | -2.64 |
|  |  | MGF | ***QPH.caas-4BS*** | 4B | AX-94555602-AX-94395005 | 623.2-627.7 | 33.56 | 30.47 | 9.78 |
|  |  |  | ***QPH.caas-4DS*** | 4D | AX-111486136-AX-110518080 | 134.5-141.3 | 10.09 | 7.19 | -4.74 |
|  |  |  | ***QPH.caas-4DS*** | 4D | AX-111071805-AX-109665328 | 175.2-185.3 | 33.82 | 35.73 | -10.39 |
|  | Ground |  | ***QPH.caas-4BS*** | 4B | AX-94555602-AX-94395005 | 623.2-627.7 | 39.31 | 30.25 | 9.87 |
|  |  |  | ***QPH.caas-4DS*** | 4D | AX-111486136-AX-110834217 | 134.5-141.3 | 8.63 | 4.61 | -3.75 |
|  |  |  | ***QPH.caas-4DS*** | 4D | AX-111071805-AX-109665328 | 175.2-185.3 | 41.17 | 36.51 | -10.64 |

^a^Quantitative trait loci

^b^ Chromosome

^c^Physical interval of SNP markers-based linkage map

^d^ LOD value of each QTL

^e^ Phenotypic variance explained by QTL

^f^ A positive sign means that positive allele comes from the parent Zhongmai 895, a negative sign means positive allele comes from the parent Yangmai 16.

Additive effect with positive and negative values indicating an increasing effect from Zhongmai 895, Yangmai 16 alleles, respectively.

B, Booting; MGF, Mid-grain filling; UAV, Unmanned aerial vehicle.

***Table S2: Details of Rht alleles across the DH population and parent cultivars***

| **Genotype** | ***Rht-B1* alleles** | ***Rht-D1* alleles** | **Genotype** | ***Rht-B1* alleles** | ***Rht-D1* alleles** | **Genotype** | ***Rht-B1*alleles** | ***Rht-D1* alleles** | **Genotype** | ***Rht-B1* alleles** | ***Rht-D1*alleles** |
| --- | --- | --- | --- | --- | --- | --- | --- | --- | --- | --- | --- |
| XY003 | *Rht-B1a* | *Rht-D1a* | XY056 | *Rht-B1b* | *Rht-D1b* | XY110 | *Rht-B1a* | *Rht-D1b* | XY164 | *Rht-B1b* | *Rht-D1a* |
| XY004 | *Rht-B1a* | *Rht-D1b* | XY057 | *Rht-B1b* | *Rht-D1b* | XY111 | *Rht-B1b* | *Rht-D1a* | XY165 | *Rht-B1b* | *Rht-D1a* |
| XY005 | *Rht-B1b* | *Rht-D1a* | XY058 | *Rht-B1b* | *Rht-D1a* | XY112 | *Rht-B1a* | *Rht-D1a* | XY166 | *Rht-B1b* | *Rht-D1a* |
| XY006 | *Rht-B1a* | *Rht-D1a* | XY059 | *Rht-B1b* | *Rht-D1a* | XY113 | *Rht-B1b* | *Rht-D1a* | XY167 | *Rht-B1b* | *Rht-D1b* |
| XY007 | *Rht-B1a* | *Rht-D1a* | XY060 | *Rht-B1a* | *Rht-D1b* | XY114 | *Rht-B1a* | *Rht-D1a* | XY168 | *Rht-B1b* | *Rht-D1b* |
| XY008 | *Rht-B1b* | *Rht-D1a* | XY061 | *Rht-B1b* | *Rht-D1a* | XY115 | *Rht-B1a* | *Rht-D1b* | XY169 | *Rht-B1a* | *Rht-D1a* |
| XY009 | *Rht-B1b* | *Rht-D1b* | XY062 | *Rht-B1a* | *Rht-D1b* | XY116 | *Rht-B1b* | *Rht-D1a* | XY170 | *Rht-B1a* | *Rht-D1a* |
| XY010 | *Rht-B1a* | *Rht-D1a* | XY063 | *Rht-B1a* | *Rht-D1b* | XY117 | *Rht-B1b* | *Rht-D1a* | XY171 | *Rht-B1b* | *Rht-D1a* |
| XY011 | *Rht-B1b* | *Rht-D1a* | XY064 | *Rht-B1b* | *Rht-D1b* | XY118 | *Rht-B1b* | *Rht-D1a* | XY172 | *Rht-B1a* | *Rht-D1b* |
| XY012 | *Rht-B1b* | *Rht-D1b* | XY065 | *Rht-B1a* | *Rht-D1b* | XY119 | *Rht-B1a* | *Rht-D1a* | XY173 | *Rht-B1a* | *Rht-D1b* |
| XY013 | *Rht-B1a* | *Rht-D1a* | XY067 | *Rht-B1a* | *Rht-D1b* | XY120 | *Rht-B1b* | *Rht-D1a* | XY174 | *Rht-B1a* | *Rht-D1a* |
| XY014 | *Rht-B1a* | *Rht-D1b* | XY068 | *Rht-B1b* | *Rht-D1a* | XY121 | *Rht-B1a* | *Rht-D1b* | XY177 | *Rht-B1b* | *Rht-D1a* |
| XY015 | *Rht-B1b* | *Rht-D1a* | XY069 | *Rht-B1b* | *Rht-D1b* | XY122 | *Rht-B1a* | *Rht-D1a* | XY178 | *Rht-B1b* | *Rht-D1a* |
| XY016 | *Rht-B1b* | *Rht-D1a* | XY070 | *Rht-B1b* | *Rht-D1b* | XY123 | *Rht-B1a* | *Rht-D1b* | XY179 | *Rht-B1b* | *Rht-D1a* |
| XY017 | *Rht-B1b* | *Rht-D1a* | XY071 | *Rht-B1a* | *Rht-D1a* | XY124 | *Rht-B1a* | *Rht-D1b* | XY180 | *Rht-B1a* | *Rht-D1b* |
| XY018 | *Rht-B1a* | *Rht-D1a* | XY072 | *Rht-B1a* | *Rht-D1a* | XY125 | *Rht-B1a* | *Rht-D1b* | XY181 | *Rht-B1a* | *Rht-D1a* |
| XY019 | *Rht-B1b* | *Rht-D1b* | XY073 | *Rht-B1b* | *Rht-D1a* | XY126 | *Rht-B1b* | *Rht-D1a* | XY182 | *Rht-B1b* | *Rht-D1b* |
| XY020 | *Rht-B1a* | *Rht-D1b* | XY074 | *Rht-B1b* | *Rht-D1b* | XY127 | *Rht-B1b* | *Rht-D1a* | XY183 | *Rht-B1a* | *Rht-D1b* |
| XY021 | *Rht-B1a* | *Rht-D1a* | XY075 | *Rht-B1b* | *Rht-D1b* | XY128 | *Rht-B1b* | *Rht-D1a* | XY184 | *Rht-B1a* | *Rht-D1b* |
| XY022 | *Rht-B1a* | *Rht-D1a* | XY076 | *Rht-B1a* | *Rht-D1b* | XY129 | *Rht-B1a* | *Rht-D1b* | XY185 | *Rht-B1a* | *Rht-D1b* |
| XY024 | *Rht-B1b* | *Rht-D1a* | XY077 | *Rht-B1b* | *Rht-D1b* | XY132 | *Rht-B1b* | *Rht-D1a* | XY186 | *Rht-B1a* | *Rht-D1b* |
| XY025 | *Rht-B1a* | *Rht-D1b* | XY078 | *Rht-B1b* | *Rht-D1a* | XY133 | *Rht-B1b* | *Rht-D1a* | XY187 | *Rht-B1a* | *Rht-D1b* |
| XY026 | *Rht-B1b* | *Rht-D1a* | XY079 | *Rht-B1b* | *Rht-D1a* | XY134 | *Rht-B1a* | *Rht-D1b* | XY188 | *Rht-B1a* | *Rht-D1b* |
| XY027 | *Rht-B1b* | *Rht-D1b* | XY080 | *Rht-B1a* | *Rht-D1b* | XY135 | *Rht-B1a* | *Rht-D1b* | XY189 | *Rht-B1a* | *Rht-D1b* |
| XY028 | *Rht-B1a* | *Rht-D1b* | XY081 | *Rht-B1a* | *Rht-D1b* | XY136 | *Rht-B1b* | *Rht-D1a* | XY190 | *Rht-B1a* | *Rht-D1b* |
| XY029 | *Rht-B1a* | *Rht-D1a* | XY082 | *Rht-B1b* | *Rht-D1b* | XY137 | *Rht-B1a* | *Rht-D1a* | XY191 | *Rht-B1a* | *Rht-D1b* |
| XY030 | *Rht-B1a* | *Rht-D1b* | XY083 | *Rht-B1b* | *Rht-D1b* | XY138 | *Rht-B1b* | *Rht-D1a* | XY192 | *Rht-B1b* | *Rht-D1a* |
| XY031 | *Rht-B1a* | *Rht-D1a* | XY084 | *Rht-B1b* | *Rht-D1a* | XY139 | *Rht-B1a* | *Rht-D1a* | XY193 | *Rht-B1b* | *Rht-D1a* |
| XY032 | *Rht-B1b* | *Rht-D1b* | XY085 | *Rht-B1a* | *Rht-D1a* | XY140 | *Rht-B1b* | *Rht-D1a* | XY194 | *Rht-B1a* | *Rht-D1a* |
| XY033 | *Rht-B1b* | *Rht-D1a* | XY086 | *Rht-B1b* | *Rht-D1a* | XY141 | *Rht-B1b* | *Rht-D1a* | XY195 | *Rht-B1a* | *Rht-D1b* |
| XY034 | *Rht-B1b* | *Rht-D1b* | XY089 | *Rht-B1b* | *Rht-D1b* | XY142 | *Rht-B1a* | *Rht-D1a* | XY196 | *Rht-B1a* | *Rht-D1b* |
| XY035 | *Rht-B1b* | *Rht-D1a* | XY090 | *Rht-B1a* | *Rht-D1b* | XY143 | *Rht-B1b* | *Rht-D1a* | XY197 | *Rht-B1a* | *Rht-D1b* |
| XY036 | *Rht-B1b* | *Rht-D1b* | XY091 | *Rht-B1b* | *Rht-D1b* | XY144 | *Rht-B1b* | *Rht-D1a* | XY198 | *Rht-B1b* | *Rht-D1a* |
| XY037 | *Rht-B1b* | *Rht-D1a* | XY092 | *Rht-B1b* | *Rht-D1b* | XY145 | *Rht-B1b* | *Rht-D1a* | XY199 | *Rht-B1a* | *Rht-D1b* |
| XY038 | *Rht-B1a* | *Rht-D1b* | XY093 | *Rht-B1b* | *Rht-D1a* | XY146 | *Rht-B1b* | *Rht-D1a* | XY200 | *Rht-B1a* | *Rht-D1a* |
| XY039 | *Rht-B1a* | *Rht-D1b* | XY094 | *Rht-B1a* | *Rht-D1b* | XY147 | *Rht-B1b* | *Rht-D1a* | XY202 | *Rht-B1b* | *Rht-D1b* |
| XY040 | *Rht-B1b* | *Rht-D1a* | XY095 | *Rht-B1b* | *Rht-D1a* | XY148 | *Rht-B1b* | *Rht-D1a* | XY203 | *Rht-B1b* | *Rht-D1a* |
| XY041 | *Rht-B1a* | *Rht-D1a* | XY096 | *Rht-B1b* | *Rht-D1a* | XY149 | *Rht-B1b* | *Rht-D1a* | XY204 | *Rht-B1a* | *Rht-D1b* |
| XY042 | *Rht-B1b* | *Rht-D1b* | XY097 | *Rht-B1b* | *Rht-D1b* | XY150 | *Rht-B1b* | *Rht-D1a* | XY205 | *Rht-B1a* | *Rht-D1b* |
| XY043 | *Rht-B1b* | *Rht-D1b* | XY098 | *Rht-B1b* | *Rht-D1a* | XY151 | *Rht-B1a* | *Rht-D1b* | XY206 | *Rht-B1a* | *Rht-D1a* |
| XY046 | *Rht-B1a* | *Rht-D1b* | XY099 | *Rht-B1b* | *Rht-D1b* | XY152 | *Rht-B1a* | *Rht-D1a* | XY207 | *Rht-B1b* | *Rht-D1a* |
| XY047 | *Rht-B1a* | *Rht-D1b* | XY100 | *Rht-B1b* | *Rht-D1a* | XY153 | *Rht-B1b* | *Rht-D1a* | XY208 | *Rht-B1a* | *Rht-D1a* |
| XY048 | *Rht-B1a* | *Rht-D1a* | XY101 | *Rht-B1b* | *Rht-D1a* | XY156 | *Rht-B1a* | *Rht-D1b* | XY209 | *Rht-B1a* | *Rht-D1b* |
| XY049 | *Rht-B1a* | *Rht-D1a* | XY102 | *Rht-B1a* | *Rht-D1b* | XY157 | *Rht-B1b* | *Rht-D1a* | XY210 | *Rht-B1a* | *Rht-D1b* |
| XY050 | *Rht-B1a* | *Rht-D1b* | XY103 | *Rht-B1b* | *Rht-D1a* | XY158 | *Rht-B1b* | *Rht-D1a* | XY211 | *Rht-B1b* | *Rht-D1b* |
| XY051 | *Rht-B1b* | *Rht-D1b* | XY104 | *Rht-B1b* | *Rht-D1a* | XY159 | *Rht-B1b* | *Rht-D1a* | XY212 | *Rht-B1a* | *Rht-D1b* |
| XY052 | *Rht-B1b* | *Rht-D1a* | XY105 | *Rht-B1b* | *Rht-D1a* | XY160 | *Rht-B1b* | *Rht-D1a* | XY213 | *Rht-B1b* | *Rht-D1b* |
| XY053 | *Rht-B1b* | *Rht-D1a* | XY106 | *Rht-B1b* | *Rht-D1a* | XY161 | *Rht-B1b* | *Rht-D1a* | YM16 | *Rht-B1b* | *Rht-D1a* |
| XY054 | *Rht-B1a* | *Rht-D1a* | XY107 | *Rht-B1b* | *Rht-D1a* | XY162 | *Rht-B1b* | *Rht-D1b* | ZM895 | *Rht-B1a* | *Rht-D1b* |
| XY055 | *Rht-B1a* | *Rht-D1a* | XY108 | *Rht-B1a* | *Rht-D1b* | XY163 | *Rht-B1b* | *Rht-D1a* |  |  |  |
